# Tropical pyomyositis as a presenting feature of subclinical leukemia: a case report

**DOI:** 10.1186/s13256-015-0513-z

**Published:** 2015-02-15

**Authors:** Mitrakrishnan Rayno Navinan, Jevon Yudhisdran, Thambyaiah Kandeepan, Aruna Kulatunga

**Affiliations:** National Hospital of Sri Lanka, Regent Street, Colombo, 10 Sri Lanka

**Keywords:** AML, Immunodeficiency, Leukemia, Tropical pyomyositis

## Abstract

**Introduction:**

Pyomyositis, though classically considered a tropical disease, has a variable geographic prevalence. Among the predisposing risk factors, immunodeficiency plays an important role. Pyomyositis has a tendency to mimic more commonly considered diseases, and a lack of familiarity with it is a cause of delayed diagnosis.

**Case presentation:**

A 53-year-old South Asian man with newly diagnosed type 2 diabetes mellitus was referred to our medical unit in an advanced stage of the disease, which was complicated by sepsis and acute kidney injury. Failure of the referring unit to provide prompt treatment, as well as their delay in coming to a diagnosis, led to the patient’s complicated state. Antibiotic therapy was initiated, and clinical stabilization was achieved with supportive measures. Following the patient’s recovery from sepsis, his persistent leukopenia and anemia was suggestive of an underlying immunodeficiency, and a subsequent bone marrow biopsy revealed acute myeloid leukemia, M2 variant. Multi-disciplinary care was initiated by the medical, surgical and oncological teams.

**Conclusion:**

Awareness of tropical pyomyositis is lacking. Common predisposing behaviors and conditions should always be sought and investigated. Immunosuppressive state is an important predisposing factor in the pathogenesis of pyomyositis. Early antibiotic treatment is pivotal in management, and surgical intervention, when relevant, should not be delayed. Identifying one cause should not halt the search for others, as pyomyositis may herald underlying sinister diseases.

## Introduction

Pyomyositis is classically considered a disease of the tropics and has been known to make up 4% of all admissions in certain African nations [1]. Immunity plays a pivotal role, as it has been observed to be commoner in patients in compromised states, whether it be secondary to HIV or to non-infective states predisposing the patient to immune system impairment (for example, diabetes mellitus, rheumatologic diseases and malignancy). This phenomenon has thus caused an increased prevalence of pyomyositis, even in temperate countries [2]. In addition to impaired immunity, other risk factors include intravenous drug use and trauma that may be considered trivial [3]. Characteristically, the predominant causative organism is *Staphylococcus aureus*, which may even be methicillin-resistant [4,5]. However, in patients in immunocompromised states, organisms may show a varying pattern, such as Gram-negative bacteria; *Streptococcus* groups B, C and G; *Mycobacterium avium* [4]; and many others. Pyomyositis mainly affects the major muscle groups of the limb girdles, especially the quadriceps, causing suppuration leading to abscesses [3,6]. The diagnosis may be difficult, as it may mimic more commonly considered diseases of the tropics, such as cellulitis, dengue, leptospirosis, viral fever and osteomyelitis [7]. This may be attributed to lack of familiarity or the absence of specific signs [3]. In this report, we present a unique case of tropical pyomyositis that heralded an underlying silent and sinister etiology masked by the presence of obvious risk factors requiring management with a multi-disciplinary approach.

## Case presentation

A 53-year-old South Asian man recently diagnosed with type 2 diabetes mellitus who had been receiving treatment for 2 months with oral hypoglycemic drugs presented to our hospital with slow, insidious, progressive swelling of the anterior aspect of the left thigh. He was otherwise healthy and denied any high-risk behavior, smoking or alcohol consumption in the past. His presentation to us was delayed, as he initially failed to seek attention with worsening symptoms. Upon his initial admission to the hospital for investigation and management, despite persistent fever, the investigating unit failed to suspect pyomyositis. They treated the patient with intravenous cloxacillin 1g every 6 hours, suspecting cellulitis. With the development of acute confusion, the patient was presented to our medical unit for further management. The patient had no preceding history of trauma. The patient’s examination revealed that he was febrile, had a high body temperature (39°C) and was confused (Glasgow Coma Scale score of 11 out of 15) with acidotic breathing. His abdominal, respiratory and remaining neurologic system examination results were normal. Despite his condition, the patient’s blood pressure remained within normal parameters, though tachycardia was noted. His left thigh was observed to be disproportionately swollen, warm and tense to touch.

Whole-blood analysis revealed a grossly elevated white blood cell count of 34×10^9^/L (normal range, 4×10^9^/L to 11×10^9^/L), which was predominantly neutrophilic (86%). He had a low hemoglobin concentration of 6.8g/dl (normal range, 11g/dl to 18g/dl) and a platelet count of 158×10^9^/L (normal range, 150×10^9^/L to 450×10^9^/L). His C-reactive protein concentration was elevated at 258mg/L (normal range, <8mg/L). His blood culture tests were negative repeatedly. His serum creatinine level was elevated at 140μmol/L (normal range, 60μmol/L to 120μmol/L) and rising, with the highest value recorded at 195μmol/L. Despite his rising creatinine level, his electrolytes remained normal. His coagulation profile was deranged, with an elevated international normalized ratio of 2.59 and an activated thromboplastin time of 41 seconds (normal range, 24 to 36 seconds). His serum ammonia level was elevated at 69μmol/L (normal range, 26μmol/L to 47μmol/L), and he had a high serum lactate dehydrogenase value of 531IU/L (normal range, 230IU/L to 460IU/L). His total bilirubin was elevated at 93μmol/L (normal range, 5μmol/L to 21μmol/L) and showed a predominant indirect fraction. His alkaline phosphatase concentration was marginally elevated at 345IU/L (normal range, 98IU/L to 279IU/L), but his aspartate and alanine transaminase values were normal at 32IU/L (normal range, <34IU/L) and 38IU/L (normal range, 11IU/L to 50IU/L), respectively. His total proteins were found to be mildly reduced at 5.1g/dl (normal range, 6.4g/dl to 8.4g/dl). Though marginally low, his albumin level remained predominant, with a value of 2.7g/dl (normal range, 3.7g/dl to 5.4g/dl). His preliminary fasting blood sugar values were found to be elevated at 215mg/dl (normal range, less than 126mg/dl), reflecting poor control.

**Figure 1:**
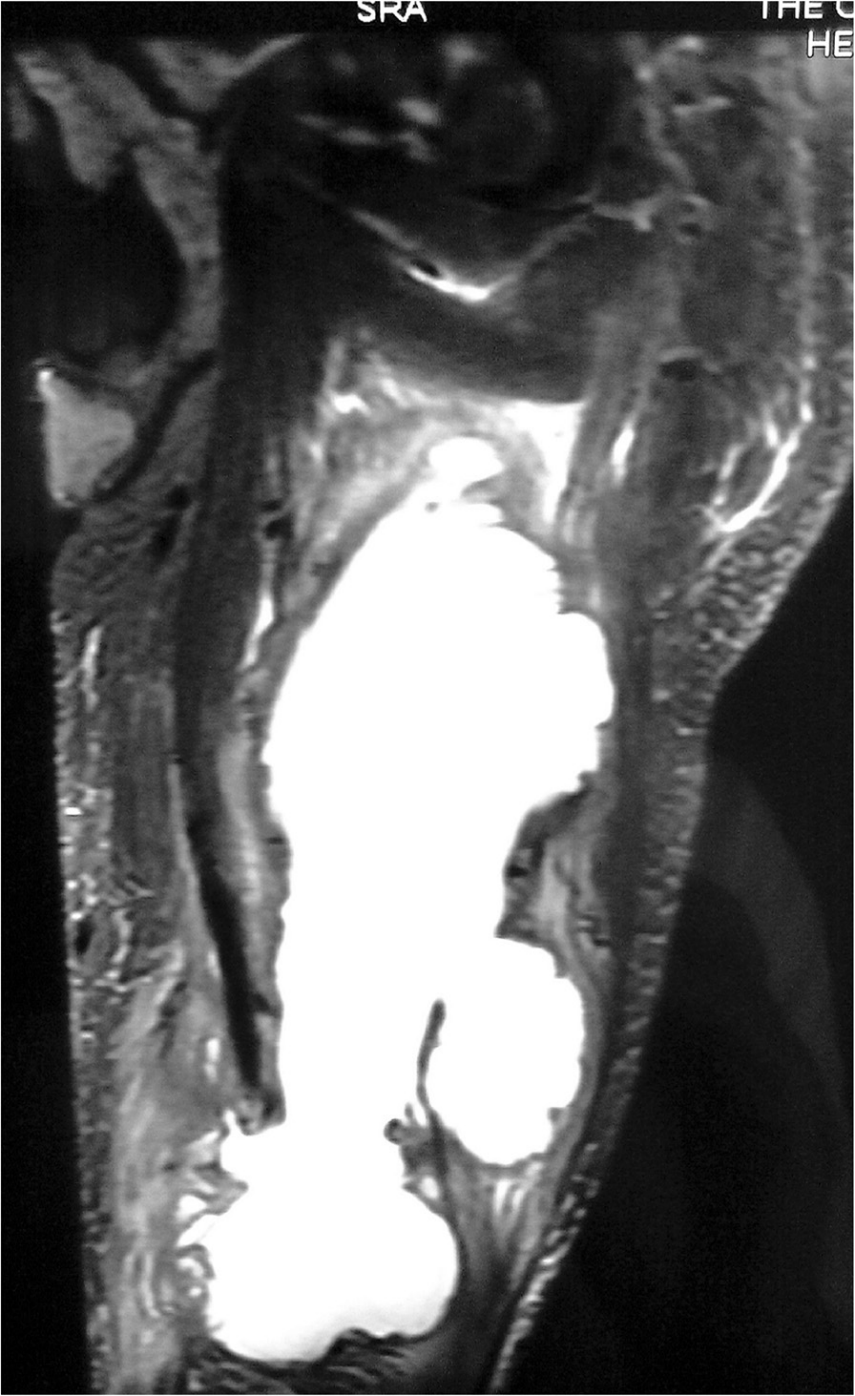
**T2-weighted magnetic resonance image of the left thigh shows pyomyositis.** T2-weighted magnetic resonance image of the left thigh taken in the sagittal plane reveals a multi-loculated mass in the medial aspect that demonstrates high signal intensity.

His blood picture revealed normocytic normochromic red cells, polychromatic cells, red cell fragments with toxic neutrophils and mild thrombocytopenia. These features were compatible with hemolysis secondary to sepsis. The result of retroviral screening was negative. Ultrasound imaging of the abdomen revealed a slightly hypoechoic, mildly enlarged liver with normal ducts and splenomegaly of 13.36cm. Magnetic resonance imaging (MRI) of the patient’s left thigh and pelvis revealed a multi-loculated mass, most likely an abscess, which measured 20cm in length and 8mm in its widest diameter. The mass demonstrated high signal intensity in the medial aspect of the thigh on T2-weighted images (Figure 1), confirming our clinical suspicion of pyomyositis. Cultures of the drained pus were found to be sterile. However, a muscle biopsy and culture was positive for a non-lactose fermenting coliform organism (sensitive to cefoperazone and sulbactam, netilmicin and ciprofloxacin; intermediate sensitivity to ticarcillin-clavulanate; and resistance to imipenem, meropenem, amikacin, ceftazidime and gentamicin). A coagulase-negative, methicillin-resistant *Staphylococcus aureus* isolate was also detected, but contamination was considered likely.

Thus, the patient was initiated on intravenous cefoperazone and sulbactam 1g every 12 hours for 2 weeks. In view of his rising serum creatinine level, we opted for one cycle of hemodialysis with strict fluid management, which resulted in resolution of his acute kidney injury. He was transfused with packed red blood cells to achieve and maintain his hemoglobin levels. He demonstrated dramatic clinical improvement, with normalization of consciousness and stabilization of his whole-blood count values to normal. After 8 days of treatment and achievement of stabilization, the significant amount of pus necessitated surgical intervention. Thus, a surgical consult was sought. The patient’s loculated pus was drained, which was found to be sterile when cultured. Tight glycemic control was achieved with subcutaneous administration of soluble insulin, and the patient’s capillary blood sugar values were maintained below 200mg/dl. However, following drainage and treatment with intravenous antibiotics, the development and persistence of leukopenia (2.51×10^9^/L (normal range, 4×10^9^/L to 11×10^9^/L)) with lymphocytic predominance of 81% (normal range, 20% to 40%), coupled with normocytic normochromic anemia in whole-blood analysis despite clinical improvement, prompted further investigation. On the basis of suspecting an underlying leukemia, a bone marrow biopsy was done and revealed a markedly hypercellular marrow with 22% abnormal blasts and reactive granulocytic hyperplasia. The flow cytometric and morphologic findings were compatible with acute myeloid leukemia (AML), M2 variant (AML-M2). The patient was then referred for further oncologic management.

## Discussion

South Asian countries such as India and Sri Lanka are classified as tropical; however, only a few sporadic cases and case series of tropical pyomyositis have been reported [8-10]. This may be due to an overall lack of awareness and underreporting rather than the lack of prevalence. In this report, we present a case of a patient with pyomyositis who demonstrated classic etiologic predisposing factors: underlying subclinical AML with type 2 diabetes mellitus that progressed to systemic sepsis with acute kidney injury. The unique presenting manifestation of AML in our patient was pyomyositis due to secondary infection.

Immunocompromised states should always be excluded when pyomyositis is suspected [3]. Although the tropical environment by itself is a risk factor, South Asians also have a high incidence of type 2 diabetes mellitus, a well-known cause of impaired immunity and a risk factor for pyomyositis. Our patient had type 2 diabetes mellitus. Though it was a recent diagnosis, it would have been a silent yet established predisposing etiologic factor. What made the case unique, however, was his dual pathology of AML-M2, which further enhanced the effect of his immunocompromised state. This might explain the large amount of pus with its complexity and loculation, and even the late stage of presentation. This case reemphasizes the need to carry out all relevant investigations to exclude underlying causes of immunocompromise. The identification of one predisposing cause should not stop the etiologic search for others, because underlying subclinical sinister causes may be discovered that require urgent intervention. The importance of immunocompetence and its impact on the presentation of pyomyositis is demonstrated by our present case. Hematologic malignancies are known to be associated with pyomyositis. Falagas and colleagues [11], in their comprehensive review, detailed the various malignancies that have been reported thus far. These include AML, acute lymphoblastic leukemia, acute myelomonocytic leukemia, chronic myeloid leukemia, chronic lymphocytic leukemia, myeloproliferative disease, myelodysplastic syndrome, plasma cell leukemia, myelomonocytic leukemia, multiple myeloma, non-Hodgkin’s and Hodgkin’s lymphoma, diffuse large B-cell lymphoma and lymphoblastic lymphoma. Though *Staphylococcus aureus* predominated in most of these documented cases, a variety of atypical organisms were also noted.

Treatment of pyomyositis depends on staging, which is categorized into three stages [3,6]. Stage 1 (invasive stage) is early and has relatively mild signs and symptoms, with minimal systemic signs and little or no pus. Stage 2 (suppurative stage) is an advanced stage in which most present, and the symptoms are severe due to presence of pus. Stage 3 (late stage) is the final stage, where there is systemic toxicity, dissemination of infection and organ dysfunction. Only the preliminary stage may be managed medically; the latter two require a combination of medical management with surgical intervention [3,6]. The case of our patient demonstrates the typical insidious pattern that progresses to purulent collections, also described by Patel and colleagues [12]. Though our patient was in the latter part of stage 2 when he sought treatment initially, his condition deteriorated quickly to stage 3. He was referred to us in a state of sepsis complicated by confusion and acute kidney injury and without a definitive diagnosis. After we made a confident diagnosis based on the patient’s history and physical signs and affirmed by MRI, the patient was hemodialyzed and his septic state was stabilized, and he was referred back for drainage of the loculated pus collection with follow-up oncologic intervention.

Tropical pyomyositis can be a disease with multiple clinical complexities. The presence of it should alert the clinician to be wary of conditions that predispose individuals to such a disease. Tropical pyomyositis may be the first heralding feature of yet to be diagnosed clinical conditions that may be more sinister than the disease process itself.

## Conclusion

Awareness of tropical pyomyositis is lacking among the medical fraternity, and a high degree of clinical suspicion should facilitate early diagnosis. Though antibiotic treatment is pivotal in the management of pyomyositis and needs to be initiated early surgical intervention, when deemed clinically relevant, should be sought. Immunosuppression plays a key role in the pathogenesis of pyomyositis, and a full spectrum of investigations to exclude common predisposing conditions should always be carried out. The identification of one cause should not halt the search for others, as rarely tropical pyomyositis may herald sinister diseases that are subclinical. Thus, typical risk factors and behaviors should be sought for and investigated, and, through the course of the illness, complications should be anticipated and managed promptly.

## Consent

Written informed consent was obtained from the patient for publication of this case report and any accompanying images. A copy of the written consent is available for review by the Editor-in-Chief of this journal.

## Abbreviations

AML: Acute myeloid leukemia
MRI: Magnetic resonance imaging
